# Postoperative Mobilization Regimens Following Digital Nerve Repair: A Systematic Review

**Published:** 2014-01-17

**Authors:** Shehab Jabir, Fortune C. Iwuagwu

**Affiliations:** St Andrews Centre for Plastic Surgery and Burns, Broomfield Hospital, Chelmsford, United Kingdom

**Keywords:** digital nerves, mobilization, regimen, repair, systematic review

## Abstract

**Introduction:** Currently there is a multiplicity of postoperative mobility-based rehabilitation protocols following isolated digital nerve repair. The regime chosen appears to be dependent on the preference of the surgeon and unit rather than being evidence based. We aim to systematically review the current evidence to provide an insight toward formulating guidelines for best practice. **Methods:** The study was carried out in accordance to the PRISMA statement for systematic reviews. Medline, Embase, CINAHL, Google Scholar, and Cochrane databases were searched from inception to June 2013. Key search terms used were as follows: “digital nerve,” “rehabilitation,” “mobilization/mobilization,” “immobilization/immobilization,” “splinting,” “non-splinting,” “brace,” “repair,” and “coaptation.” **Results:** Four studies met the inclusion criteria and compared 2 of 3 regimens: complete immobilization, protected mobilization, and free mobilization. The primary outcome measured sensibility via 2-point discrimination and Semmes-Weinstein monofilament testing. There was no statistically significant difference in sensibility between either of the regimens. Secondary outcome measures included subjective measures such as cold intolerance and hyperesthesia, which also showed no significant difference between protocols. One study found that stiffness was increased, and return to work delayed, when a splinting protocol was employed. **Conclusions:** Current evidence suggests that all 3 protocols are equivalent in their outcomes. The stiffness and delayed return to work associated with splinting protocols indicate that free mobilization protocols may have an advantage over them. However, the limitations of current evidence mean that the hand surgeon and therapist should choose a regimen from those discussed earlier, which is tailored to the needs of each individual patient until further evidence is gathered.

Digital nerve injuries are the most common type of nerve injury in the upper limb.^1^ Despite its frequency and almost a century of research in this field, peripheral nerve repair has not resulted in achieving a consistently excellent level of function after repair.^2^^,^^3^ The microsurgical methods of repair are well described, and currently no further refinements to improve outcomes of digital nerve repair within this sphere can be envisaged. Thus current research has placed emphasis on biological manipulation of neural regeneration in an attempt to improve outcomes following coaptation of injured nerves.^4^ Little attention has, however, been paid to postoperative mobilization regimens following digital nerve repair. Currently there is much controversy, with the postoperative mobility regimen, usually decided by the attendant surgeon or surgical unit in an empirical way.

Two studies using cadaveric models of digital nerve repair, where various segments of nerves were resected and then repaired, have shown that as long as the resected segments of digital nerves are less than or equal to 2.5 mm they can be mobilized freely with no risk of rupture at the site of repair.^5^^,^^6^ A study by Lee et al (1999) comparing the histological changes associated with postoperative mobilization following median and ulnar nerve repair in a canine model suggests that early mobilization impedes nerve regeneration by delaying revascularization and enhancing scar formation.^7^ The extrapolation of these results to the clinical setting in humans may be restricted by the canine nature of the species used in the study; however, they do suggest that splinting and immobilization following nerve repair may be necessary in certain circumstances to encourage successful nerve regeneration at the repair site. On the contrary, splinting/immobilization could lead to joint stiffness and impaired tendon gliding, which might affect functioning of the digit and delay in return to work.^8^ Hence there is a need for clear guidance on the ideal regimen, which would balance the need for integrity of the nerve repair in the immediate postoperative period, while at the same time preventing the deleterious consequences of immobilization on function of the digit.

In this study, we aim to undertake a systematic review of the literature on postoperative mobility-based rehabilitation following primary digital nerve repair to determine the ideal postoperative regimen for this injury. It is thought that this is a necessary and important undertaking due to several reasons:

1. The current controversy on the topic with no clear evidence of the superiority of immobilization over mobilization or vice versa.
2. The high incidence of digital nerve injuries.^1^
3. The potential for these injuries to result in significant functional impairment following suboptimal postoperative rehabilitation.
4. The economic impact of splinting and its deleterious sequelae in terms of cost to the provider and the patient.^8^

## METHODS

The systematic review was performed in accordance to the guidelines set out in the PRISMA statement.^9^^,^^10^ Items 12 to 16 of the PRISMA statement were not applicable to this study as no quantitative data synthesis was undertaken.

### Protocol and registration

The systematic review protocol was formulated and registered with the International prospective register of systematic reviews (Registration number: CRD42012002487; available from http://www.crd.york.ac.uk/prospero/display_record.asp?ID=CRD42012002487).

### Eligibility/inclusion criteria

The criteria listed in Table 1 were used to determine eligibility of a study to be included in the systematic review.

### Information sources and search strategy

A literature search was carried out on Medline, Embase, CINAHL, Google Scholar, and the Cochrane from inception of the database to June 2013 for studies on the topic of postoperative mobility regimens following digital nerve repair. The following key words were used: “digital nerve,” “rehabilitation,” “mobilization/mobilization,” “immobilization/immobilization,” “splinting,” “non-splinting,” “brace,” “repair,” “coaptation,” and “neurorrhaphy.” The search terms were combined with the Boolean operator “and.” The references of selected studies were also perused for articles that may have been missed via the electronic search.

### Study selection

The title and abstract of all identified studies were examined by one reviewer (S.J.). In cases where suitability of a study for inclusion in the review was unclear, the entire article was obtained and assessed for suitability. Eligibility as mentioned earlier was determined by the criteria listed in Table 1. Any issue pertaining to eligibility of studies was solved via discussion with the senior author (F.I.).

### Data collection process and data items

Data were extracted onto a Performa, which included the data categories listed in Table 2.

## RESULTS

### Study selection

**Figure 1:**
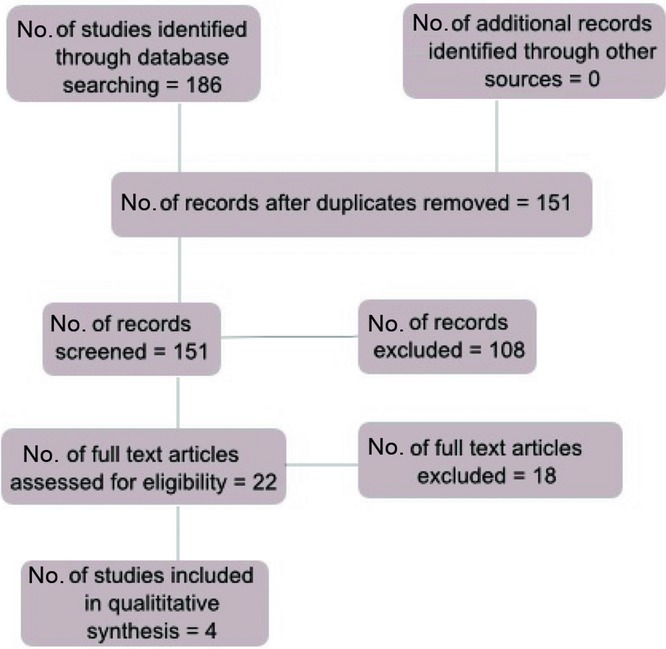
Flow diagram of search strategy.

The search retrieved a total of 122 studies following removal of duplicates. A total of 104 studies were excluded following screening of the title and abstract. The entire manuscripts of the remaining 18 articles were reviewed to establish suitability for inclusion. Fourteen studies were excluded as they did not meet the eligibility criteria leaving 4 studies for inclusion in the review (see Fig 1).

### Study characteristics

#### Study design

The 4 studies consisted of a mixture of study designs as shown in Table 3.^4^^,^^8^^,^^11^^,^^12^

#### Demographic characteristics

There were 138 patients in all 4 studies, distributed among 3 postoperative mobility protocols: mobilization, protected mobilization, and immobilization. Each study incorporated a design where 2 of these 3 protocols were compared. The total number of patients in each of these 3 protocols is given in Table 4, whereas demographic characteristics of each of the 2 groups in each study are given in Table 5 (for further elaboration on mobility protocols used within each study, please refer to the section “Postoperative Characteristics”). All 4 studies used well-matched study groups in terms of age and gender apart from Clare et al (2004) who had a 7-year gap between the splinted and unsplinted groups (see Table 5).

#### Preoperative characteristics

Apart from Yu et al (2004), all other studies recruited only those with uncomplicated sharp transection of digital nerves for both the splinted and unsplinted groups (Table 6). Yu et al recruited patients with uncomplicated sharp transection of digital nerves for the splinted group and those with concomitant sharp digital nerve laceration and flexor tendon repair for the unsplinted group.

#### Intraoperative characteristics

All patients had repair of the transected digital nerve under a microscope with 8-0, 9-0, or 10-0 nonabsorbable epineural suture (Table 6). Vipond et al (2007) did not provide information on the suture used; however, it would not be unreasonable to assume that they used one of those mentioned earlier as these nerve repair protocols are well standardized.

#### Postoperative characteristics

Following digital nerve repair, the patients underwent 1 of 3 postoperative rehabilitation protocols as in Table 5. They were then followed-up at various time points as shown in Table 7. The assessments included 2-point discrimination (2 PD—static ± dynamic), Semmes-Weinstein monofilament, and range of motion as shown on Table 8. Direct comparison of the results between studies is not feasible, as certain studies did not provide the mean values for these tests (eg, Yu et al [2004] and Vipond et al [2007]). Some of the studies also attempted to gather information on cold intolerance, hyperesthesia, and stiffness on a Likert scale as well as time to return to work measured in number of days (Table 9). Again direct comparison of these subjective outcomes between studies cannot be made as certain studies did not use these outcomes or did not provide them in a format that enabled comparison (eg, Yu et al [2004]).

## DISCUSSION

This systematic review of the impact of postoperative rehabilitation protocols on digital nerve recovery following digital nerve repair has shown us that there are currently 3 applied protocols. It is therefore a misnomer to divide the protocols into the categories of “splinted” and “unsplinted” as is the case with the studies reviewed and in common clinical practice in several specialist units that treat this condition. This is because with splinting, there is both a protected mobilization as well as an immobilization protocol.

The primary outcome used to measure digital nerve recovery following digital nerve repair was sensibility (Table 8), which is assessed by static and dynamic 2-point discrimination, and Semmes Weinstein monofilament testing. In this review, it was not possible to combine the data from all 4 studies on these 2 measures as in some cases the means were not provided. The effect of the various rehabilitation protocols used are summarized below:

– Yu et al (2004) found no statistically significant difference between protected mobilization and complete immobilization on static 2 PD and SWM testing.
– Clare et al (2004) found no statistically significant difference in static or dynamic 2 PD and SWM between free mobilization and protected mobilization.
– Vipond et al (2007) also found no statistically significant difference in static 2 PD and SWM between free mobilization and protected mobilization.
– Finally, Henry et al (2011) found no statistically significant difference in static and dynamic 2 PD (they did not use SWM testing) between protected active mobilization and complete immobilization.

Apart from recovery of sensibility, some studies also investigated the effect of the various regimens on subjective outcomes such as cold intolerance, hyperesthesia, scar sensitivity, and stiffness (Table 9). In the study by Vipond et al (2007), both groups had comparable degrees of cold intolerance and hyperesthesia at 6 months follow-up, whereas in the study by Henry et al (2011) both groups had comparable degrees of scar sensitivity and cold intolerance at 6 to 18 months’ follow-up. Clare et al (2004) found that the group who were allowed to mobilize their digit freely returned to work significantly quicker and also had a significantly lesser degree of stiffness and cold intolerance than the group who had protected mobilization of their injured digit.

In addition, other factors such as range of motion, stiffness, and time to return to work are important considerations when deciding which postoperative rehabilitation protocol should be applied to a patient. In this regard, as stated earlier, Clare et al (2004) advocates free mobilization as superior to protected mobilization (though range of motion itself was not measured in the study). However, Yu et al (2004) found no difference in the range of motion, whereas Henry et al (2011) found no difference in the range of motion and return to work, between digits with complete immobilization and those with protected mobilization.

There are clearly limitations in this study. These include the small number of studies found which addressed the question of this review and the inadequate power in all 4 studies that were included. The reader may question as to why Yu et al, who used a group with digital nerve transection combined with flexor tendon repair, was included in this review. We would like to point out that this group underwent an immobilization regimen. Hence the inclusion of the flexor tendon repair in this group is of no consequence to the final outcomes in terms of digital nerve repair.

## CONCLUSIONS

There is just not enough high-level evidence to compare the different treatment options and their many confounding variables that make scientific analysis difficult. The literature contained insufficient details to enable us make an acceptable recommendation on any particular mobilization protocol after digital nerve repair. However, on the basis of these few studies that meet our inclusion criteria, we can speculate that current published knowledge on the topic of postoperative mobility/splinting shows no real advantage in terms of digital nerve recovery and sensibility in any 1 of the 3 postoperative rehabilitation regimens with 3 of them appearing more or less equivalent and resulting in a return of sensation in keeping with expected outcomes following digital nerve repair.^2^^,^^13^^,^^14^ On the other hand, it appears that a regimen that enables mobilization of the finger reduces postoperative stiffness and speeds up return to work. Hence, it could be argued that as all 3 regimens produce more or less the same outcome in terms of the return of sensibility, the choice of regime should probably be determined by the resultant postoperative stiffness it is likely to cause, with stiffness being reduced in those regimes that enable movement of the operated finger. Therefore, using a free mobilization regime has a real advantage in this respect. Furthermore, the benefits of early mobilization following surgery or injury to the hand are well documented, which we believe provides further support to this view. Moreover, it could be argued that postoperative inflammation and swelling of the digit provides a “natural” means by which the finger is “splinted” anyway reducing the need for artificial splinting of the digit postoperatively.

Apart from mobility regimens, other forms of postoperative rehabilitation techniques such as sensory reeducation via tactile stimulation may also help the patient recover sensibility and better digital function.^15^ This is another potential option the hand surgeon and therapist may wish to consider.

Future research should attempt to make comparisons between all 3 regimens (protocols) instead of restricting themselves to 2 regimens only. Apart from sensation, which is the primary outcome measure recorded via Semmes-Weinstein monofilament testing and 2-point discrimination, other factors such as stiffness, range of motion, number of sutures placed, associated digital artery lacerations on the injured site, location or site of those digital nerve injuries (in relation to the joints), attrition rate in the final follow-up, return to work, and the cost of each of the regimens (which are not as meticulously recorded in most of the earlier reviewed studies) are all essential. Cost is vital as it may greatly impact on the adherence of a patient to a particular regimen. Ultimately, it is a multicenter randomized control trial design incorporating the aforementioned factors that will provide evidence for the best regimen.

## Figures and Tables

**Table 1:** Predetermined inclusion and exclusion criteria for this study

Inclusion criteria
Human studies
English language
Primary digital nerve repair following sharp transection of nerve
Clearly described postoperative rehabilitation protocol
Digital nerve repair as the primary outcome measure
Exclusion criteria
Outcome of other injures i.e. tendon/vessel as the primary purpose of the study
Cadaver models
Digital nerve of the foot

**Table 2:** Categories for data extraction from selected articles

Demographic
Mean age
Gender
Hand dominance
Occupation
Preoperative
Type of injury
Sharp laceration
Crush
Avulsion
Intraoperative
Type of repair
Postoperative
Rehabilitation protocol employed
Objective outcome
Static 2 PD
Dynamic 2 PD
SMW
ROM
Subjective outcome
Cold intolerance
Hyperesthesia
Stiffness
Return to work

2 PD indicates 2-point discrimination; ROM, range of motion; SMW, Semmes-Weinstein monofilament.

**Table 3:** Study design employed in each of the studies in this review

Study	Design
Yu et al	Retrospective review
Clare et al	Blinded cohort study
Vipond et al	Randomized controlled trial
Henry et al	Retrospective review

**Table 4:** Total number of patients in each protocol

Mobility protocol	Total number of patients
Mobilization	34
Protected mobilization	70
Immobilization	36

**Table 5:** Protocols employed, number of patients in each protocol group, average age, male to female ratio, and hand dominance in each study

	Protocol employed, No. patients (average age)				
Study	Free mobilization	Protected mobilization	Immobilization	Male: Female	Injured hand: dominant/nondominant
Yu et al		14 (45)	12 (44)	18:8	11/15
Clare et al	20 (36)	20 (43)		26:14	Not available
Vipond et al	14 (30.3)	12 (30.2)		20:6	18/8
Henry et al		24 (31)	22 (33)	29:17	Not available

**Table 6:** Intraoperative characteristics of patients in each group and digital nerve repair technique

	Type of injury			
Study	Free mobilization	Protected mobilization	Immobilization	Repair
Yu et al		Uncomplicated sharp transection of nerve	Nerve transection combined with flexor tendon repair	9–0 or 10–0 nonabsorbable epineural suturing
Clare et al	Uncomplicated sharp transection of nerve	Uncomplicated sharp transection of nerve		8–0 or 9–0 nonabsorbable epineural suturing
Vipond et al	Uncomplicated sharp transection of nerve	Uncomplicated sharp transection of nerve		Epineural suture; no other details
Henry et al		Uncomplicated sharp transection of nerve	Uncomplicated sharp transection of nerve	8–0 or 9–0 nonabsorbable epineural suturing

**Table 7:** Length of follow-up at which point data were gathered from each group

	Mean follow-up		
Study	Free mobilization	Protected mobilization	Immobilization
Yu et al		42 mo	51 mo
Clare et al	21 mo	20 mo	
Vipond et al	3 and 6 mo	3 and 6 mo	
Henry et al		Between 6–18 mo	Between 6–18 mo

**Table 8:** Postoperative objective outcomes measures

	Free mobilization				Protected mobilization				Immobilization			
Study	Mean static 2 PD	Mean dynamic 2 PD	Mean SMW	ROM	Mean static 2 PD	Mean dynamic 2 PD	Mean SMW	ROM	Mean static 2 PD	Mean dynamic 2 PD	Mean SMW	ROM
Yu et al					…	…	…	Full ROM	…	…	…	Full ROM
Clare et al	5.8	…	1.6	…	5	…	1.8	…				
Vipond et al	…	…	…	…	…	…	…	…				
Henry et al					6.8	5	…	Full ROM	6.4	4.8	…	Full ROM

2 PD indicates 2-point discrimination; ROM, range of motion; SMW, Semmes-Weinstein monofilament.

**Table 9:** Postoperative subjective outcome measures

	Free mobilization				Protected mobilization				Immobilization			
Study	Cold	Hyperesthesia	Stiffness	Mean days to return to work	Cold	Hyperesthesia	Stiffness	Mean days to return to work	Cold	Hyperesthesia	Stiffness	Mean days to return to work
Yu et al					…	…	…	…	…	…	…	…
Clare et al	…	…	…	12	…	…	…	37				
Vipond et al	8	8	…	…	11	11	…	…				
Henry et al					10	…	…	50	10	…	…	55
